# Barriers to Walking: An Investigation of Adults in Hamilton (Ontario, Canada)

**DOI:** 10.3390/ijerph13020179

**Published:** 2016-01-30

**Authors:** Andrew F. Clark, Darren M. Scott

**Affiliations:** 1 Human Environments Analysis Laboratory, Department of Geography, University of Western Ontario, 1151 Richmond St., London, ON N6A 3K7, Canada; aclark2@uwo.ca; 2 Transportation Research Lab (TransLAB), School of Geography and Earth Sciences, McMaster University, 1280 Main St. West, Hamilton, ON L8S 4K1, Canada

**Keywords:** binary logit model, barriers, active transportation, walking, time availability, walkability, built environment, social environment

## Abstract

This study investigates perceived barriers to walking using data collected from 179 randomly-selected adults between the ages of 18 and 92 in Hamilton, Ontario, Canada. A survey (Hamilton Active Living Study) asked questions about socio-demographics, walking, and barriers to walking. A series of binary logit models are estimated for twenty potential barriers to walking. The results demonstrate that different barriers are associated with different sub-groups of the population. Females, senior citizens, and those with a higher body mass index identify the most barriers to walking, while young adults, parents, driver’s license owners, and bus pass owners identify the fewest barriers. Understanding who is affected by perceived barriers can help policy makers and health promotion agencies target sub-groups of the population in an effort to increase walking.

## 1. Introduction

Over the past decade, researchers have sought to understand factors that influence the decision to walk. Walking is important to understand as it can help mitigate health problems, such as asthma, diabetes, heart disease, obesity, and some forms of cancer [1], which cost taxpayers billions of dollars every year [1,2]. Walking is one of the most popular forms of physical activity [3,4,5], but there is still much to be learned about how perceived barriers make walking difficult.

Despite walking being one of the more popular forms of physical activity and highly recommended by health promotion organizations [6], few people use active modes of travel on a regular basis. For example, walking as a mode of travel is only used by 36% of Canadians to get to a routine destination on a regular basis [7]. This low modal share combined with the many benefits that walking has on both the environment and human health, has made walking a focus for researchers and policy makers [8,9]. Understanding how factors, such as the built environment, social environment, and safety, are related to walking provides researchers and policy makers the opportunity to recommend policy changes that work to minimize or enhance the effect factors have on the decision to walk.

Walking has been investigated extensively by researchers in order to gain a better understanding of the factors related to increasing and decreasing walking. Reviewing the literature finds five themes significantly related to walking: the built environment, the social environment, meteorology, safety, and topography. The built environment is the factor most frequently found to influence walking in the literature. A recent review by Brownson *et al.* [10] identifies four measures of the built environment significantly related to walking: population density, land use mix, street connectivity, and sidewalk availability. The walkability index developed by Frank *et al.* [11], which combines population density, land-use mix, street connectivity, and retail floor area ratio into a single index, is also consistently found to be related to walking [11,12,13,14].

The aesthetics of a neighborhood also play a role in the decision to walk, where unappealing neighborhoods make it less desirable for residents to walk [15]. Friends and family members can increase an individual’s walking as walking becomes a social experience rather than simply travel [15,16,17,18]. The weather also plays a part in the decision to walk [19,20,21,22]. Extreme temperatures, precipitation, and high winds discourage people from walking. The safety of a neighborhood is another factor influencing walking [23,24,25,26], as neighborhoods with higher incidences of crime and those with more dangerous streets discourage people from walking. Finally, living in a neighborhood that is hilly or has steep streets has been found to decrease walking [18,23,26,27]. If topography makes walking too treacherous or difficult, people will avoid walking.

Researchers have also examined perceived barriers to walking [28,29,30], which focus primarily on general physical activity. Barriers refer to the environmental and personal obstacles that discourage physical activity. Researchers have used the concept of barriers to determine what prevents certain sub-groups of the population from participating in physical activity, such as walking. Spinney and Millward [30] examine the extent income and time poverty act as barriers to regular participation in moderate or higher intensity physical activity among Canadian adults. The results find that time poverty is a more important variable than income poverty for engaging in regular physical activity. Work by Dawson *et al.* [29] examines the demographic factors associated with barriers to walking for middle-aged and older U.K. adults. Findings show that citing more than one environmental barrier to walking significantly decreases walking. They also find health problems affect walking more than perceived barriers. Adachi-Mejia *et al.* [28] identify perceived intrinsic barriers to physical activity among mothers in rural areas. Results find that lack of self-discipline, lack of time, and lack of interest are the most significant perceived barriers to physical activity participation.

This study builds on these past works to examine how different sub-groups of the population are associated with potential barriers to walking. To achieve this objective, this study uses data from the Hamilton Active Living Study (HALStudy) collected from May to September 2010 in Hamilton, Ontario, Canada. From these data, a set of 20 binary logit models are used to determine which sub-groups are affected by each potential barrier. Each binary logit model uses a different barrier to walking as the dependent variable, comparing those who agree that the barrier prevents them from walking more often (1) with those who disagree (0). Socio-demographics are included to identify sub-groups associated with each potential barrier. A walkability index is included in the models to examine the influence that the neighborhood has on each potential barrier.

The next section describes the data collection process and the method of analysis used for this study. The results section discusses which sub-groups agree more often with the potential barriers to walking. Finally, the conclusion summarizes the key findings and discusses their importance in the context of the literature.

## 2. Experimental Section

### 2.1. Data

The dataset used for this study is from the HALStudy, collected from May to September in 2010 after receiving institutional ethics approval for the protocol (Grant Number: 410-2008-0820). The HALStudy is a multi-instrument survey that examined the active lifestyle of the adult population in Hamilton, Ontario, Canada. Subjects were recruited by cold-calling residents in 24 neighborhoods throughout the city. Several instruments were used in the HALStudy data collection: a training interview that collected height and weight, and a perceptual neighborhood drawing; a personal questionnaire; passive-GPS tracking for seven consecutive days; and a seven-day time-use diary. A full description of the data collection methodology can be found elsewhere [31]. All participants signed letters of informed consent before participating in the survey.

This study uses the data collected from the personal questionnaire on active living. The personal questionnaire is a detailed survey examining different types of physical activity, and includes questions about participation, perceptions, and potential barriers to types of physical activity. Additional questions were asked about perceptions of physical activity, perceptions of neighborhoods, travel behavior preferences, and socio-demographics.

For this study, the focus is on barriers to walking. The data come from the walking and socio-demographic sections of the personal questionnaire. The final sample size is 179 participants that completed both the survey and the time-use diary. Each model has a sample size determined by the number of people who either agree or disagree with the barrier being modeled, as subjects who did not answer the question or stated they neither agreed nor disagreed are not modelled. The sample size ranges from 44 to 166 with an average of 137 subjects answering agree or disagree to the perceived barrier questions.

### 2.2. Concepts and Measures

#### 2.2.1. Dependent Variable

The dependent variables are binary measures of the twenty barriers to walking questions examined in this study. Each question is based on the statement *“It is difficult for me to walk more often because...”* with the answers given using a five-point likert-scale. The likert-scale answers include strongly agree, somewhat agree, neither agree nor disagree, somewhat disagree, and strongly disagree. The list of the perceived barriers to walking questions is found in Table 1. Binary variables are created for each barrier; a value of one represents agreement with the barrier (strongly and somewhat agree) and a value of zero indicates disagreement with the barrier (strongly and somewhat disagree). Missing values and those who neither agree nor disagree are removed from the analysis to allow a direct comparison of those who agree to those who disagree to each barrier.

**Table 1:** Descriptive analysis of perceived barriers to walking.

It is difficult for me to walk more often because…	Sample Size	% Disagreed	% Agreed
Accessibility			
Work is too far away **^a^**	84	48.8%	51.2%
School is too far away **^b^**	49	59.2%	40.8%
Other destinations are too far away	136	62.5%	37.5%
Appearance Concerns			
I don’t like to get hot and sweaty	141	80.9%	19.1%
I don’t like being exposed to the sun	146	74.0%	26.0%
Exercise Saturation			
I get plenty of exercise by other means	138	57.2%	42.8%
I walk enough already	135	74.8%	25.2%
I walk too much already	139	94.2%	5.8%
Health			
I have a physical disability that affects my ability to walk	166	78.9%	21.1%
I have an injury that affects my ability to walk	152	75.7%	24.3%
I’m too old	156	99.4%	0.6%
I’m not in good health	153	81.7%	18.3%
Neighborhood Safety			
I don’t have a safe place to walk because of crime	155	89.7%	10.3%
I don’t have a safe place to walk because of stray dogs	157	94.9%	5.1%
Streets have poor lighting at night	138	82.6%	17.4%
I don’t feel safe walking alone during the day	160	96.3%	3.8%
I don’t feel safe walking alone at night	156	60.9%	39.1%
Sidewalk Quality			
There are no sidewalks in my neighborhood	163	96.9%	3.1%
The sidewalks in my neighborhood are not well maintained	160	94.4%	5.6%
Time Availability			
I don’t have time	141	56.0%	44.0%
I don’t make time	145	42.8%	57.2%
I don’t have childcare assistance **^c^**	44	75.0%	25.0%
I work too much **^a^**	76	59.2%	40.8%
Walking Preference			
I don’t like to walk	146	90.4%	9.6%
I don’t feel like walking	136	75.7%	24.3%
I have no one to walk with	142	83.1%	16.9%
Routes are boring	136	79.4%	20.6%
Traffic Safety			
There is too much traffic in my neighborhood	139	83.5%	16.5%
Drivers often exceed the posted speed limits in my neighborhood	145	54.5%	45.5%
There are dangerous crossings in my neighborhood	138	73.9%	26.1%

**^a^** Questions were answered by employed individuals; **^b^** Question was answered by students; **^c^** Question was answered by parents with children.

#### 2.2.2. Independent Variables

Two types of independent variables are used in this study: individual characteristics and the built environment. A statistical summary of the independent variables is provided in Table 2. The individual characteristic variables are used to examine how sub-groups of the population are associated with perceived barriers to walking. The variables used include age, sex, household income status, marital status, student status, employment status, parenthood, driver’s license ownership, transit pass ownership, and body mass index (BMI). The explanation for choosing these variables and a definition of their specification are as follows.

**Table 2:** Summary statistics of independent variables used in the binary logit model.

Variables	Statistics
Age	
Young Adults (18–30)	16.2%
Middle Ages (31–64)	62.0%
Senior Citizens (65+)	21.8%
Female	69.3%
Income Classification	
Income Poor	14.0%
Middle Income	33.5%
Income Rich	22.3%
Missing Income	30.2%
Marital Status—Single	39.7%
Parent of Child(ren)	31.3%
Driver’s License Ownership	85.5%
Public Transit Pass Ownership	17.3%
Currently a Student	17.9%
Currently Employed	52.5%
Walkability Index, mean (s.d.)	0.000 (3.826)
Body Mass Index, mean (s.d.)	27.380 (6.851)

Age is measured by classifying continuous ages into three groups: young adults (18–30), middle-aged (31–64), and senior citizens (65 and over). These age groups are selected as the relationship between age and perceived barriers may not be linear making it important to highlight different age groups. Age is important to examine in this study as different age groups may be affected by barriers differently.

Sex is defined as a binary variable, comparing females (1) to males (0). This is important to examine as research has documented that some barriers, such as safety, are more of a concern for women than for men. There is also evidence from past research that men are more likely to use walking than women [32,33,34], highlighting the importance of examining sex differences in perceived barriers to walking.

Household income is defined in this study based on low-income cut-offs (LICO) as published by Statistics Canada [35]. LICO are defined as “income thresholds at which a family is relatively worse off compared to the average family, because it has to spend a greater portion of its income (*i.e.*, at least 20% more than average) on basic goods and services (*i.e.*, food, clothing and shelter) than the average family of similar size” [30]. High-income cut-offs (HICO) for financial wealth use a 200% increase over LICO [30] to establish three categories of wealth: HICO, LICO, and intermediate income. The classification of HICO and LICO provided by Spinney and Millward [30] are used in this study to provide an understanding of how LICO and intermediate income are associated with the barriers to walking compared to HICO.

Marital status is defined in this paper as either single (1) or married/cohabitating (0). Marital status is important to evaluate as those who live with a partner have a natural live-in companion with whom to walk. While being single does not necessarily preclude having someone to walk with, a partner who lives in the same home may increase the likelihood of walking and minimize some perceived barriers, such as safety and social influences [36,37]. Similarly, parenthood, defined as having children living in the home (1) or no children living in the home (0), provides natural live-in companions for walking, minimizing the impact of safety and social influences. Children also place time constraints on their parents making it difficult for parents to find time to walk to destinations rather than using other modes of travel.

Employment status is defined as being a full-time or part-time worker (1) or not being a worker (0), while student status is defined as being a full-time or part-time student (1) or not being a student (0). Both of these variables are included in this study for the same reason; those who have major time commitments in life, such as school or a job, have far more pressure on their time potentially leading to additional barriers.

Owning a driver’s license or transit pass (1) *vs.* not having them (0) identify who has access to various modes of travel. Those who have a license are more likely to use the car as a primary mode of travel, while those with a transit pass walk and use transit to travel. By including these variables it is possible to understand the influence travel mode has on the potential barriers to walking.

BMI is a method to evaluate the body type of individuals [37]. Those with higher BMI values are generally unhealthier and are at far higher risks of disease, such as asthma, type-two diabetes, and heart disease. Including a continuous measure of BMI in this study provides evidence as to whether those with higher BMI experience different barriers than those with lower BMI values.

The built environment is measured using a walkability index. A walkability index combines multiple measures of the built environment into a single index indicating the walkability of a neighborhood. The index used in this study is based on a 1000-m buffer around the home. The index combines population density, intersection density, retail floor area ratio, entropy index, and pedestrian infrastructure into a single value by normalizing the values for each variable and adding them together [11], with a higher value representing a more supportive walking environment. The walkability index allows us to understand how different types of neighborhoods influence potential barriers to walking.

### 2.3. Analysis Methodology

The method of analysis is a binary logit model, which is estimated for each of the twenty potential barriers to walking. The models are estimated with STATA 11.0 SE [38] to determine the propensity or likelihood that an individual will agree that each barrier influences walking behavior. The logit model was chosen for this study as it makes no assumptions about the distribution of the variables and has the ability to provide valid estimates regardless of the study design [39]. Robust cluster standard errors is added to the model to correct the standard errors that are biased due to overlapping geographies and multi-collinearity in the data [40].

The results of the models are presented using odds ratios, which are the number of those who agree with a barrier divided by the number of those who do not agree [41]. Values greater than one have an increased odds of agreeing with the barrier while values between zero and one have an increased odds of not agreeing with the barrier. Values below one can be interpreted by dividing the value of one by the odds ratio to get the reverse relationship. For example, an odds of 0.2 can be divided into 1.0 to find that for every 5 people who do not agree with a barrier there is one person who does agree [41]. Odd ratios are reported only for significant relationships (*p* < 0.10).

## 3. Results and Discussion

### 3.1. Preliminary Analysis

A descriptive analysis of the barriers to walking is shown in Table 1. Comparing the percentages of people who agree and disagree with the barriers finds the majority of people disagree that the barriers prevent them from walking more often, with the exception of *work is too far away* and *I don’t make time*. Further analysis is required to understand how these perceived barriers to walking are influenced by characteristics of the population.

### 3.2. Model Results

Although there are thirty perceived barriers to walking discussed in the preliminary analysis, only a sub-set is selected to be modeled due to issues with sample size and variability in the answers provided by the subjects. First, at least 130 subjects have to either agree or disagree with the perceived barrier to ensure at least ten observations per variable are used in the model. Second, only barriers with both agreement and disagreement above 6% are modeled to ensure that all independent variables have enough variation to be estimated. When there is less than 6% of agreement or disagreement, the models do not produce estimates because there is not enough variability in them. After filtering out barriers that did not meet the above conditions, twenty barriers are left to be modeled. The results are found in Table 3 and are discussed based on socio-demographic characteristics to determine how sub-groups of the population are associated with potential barriers to walking.

#### 3.2.1. Age

The first socio-demographic variable examined is age. Young adults are found to agree with two potential barriers compared to middle-aged adults: *destinations are too far away* and *I walk enough already*. Both barriers express the dislike young people have towards walking as they perceive distances to be long and believe minimal walking is enough.

Senior citizens are associated with five potential barriers to walking: *streets have poor lighting at night*, *too much traffic*, *dangerous crossings*, *I have no one to walk with*, and *I don’t like to walk.* The first three barriers are related to concerns about safety, as senior citizens are a vulnerable population that may not have the same strength and mobility of younger age groups. Having no one to walk with is another barrier to walking and is related to safety. The social aspect of walking provides companionship along with a feeling of security that is important to many senior citizens. The final potential barrier, *I don’t like to walk*, indicates that seniors like to walk less than middle-aged adults, possibility due to potential mobility constraints.

**Table 3:** Results of binary logit models determining characteristics of individuals associated with each barrier to walking (*p* < 0.10). Odds ratios are presented.

It Is Difficult for Me to Walk more often because…	Young Adults (18–30)	Senior Citizens (65+)	Income Poor	Middle Income	Female	Single	Student	Worker	Parent	Driver‘s License	Transit Pass	Body Mass Index	Walkability Index
Accessibility													
Destinations are too far away (excludes school and work)	2.825	-	-	-	2.043	-	-	-	-	-	-	-	-
Appearance Concerns													
I don’t like to get hot and sweaty	-	-	-	-	3.118	-	2.627	-	-	-	-	2.224	1.121
I don‘t like being exposed to the sun	-	-	-	-	3.303	0.461	-	-	-	-	-	1.763	-
Exercise Saturation													
I walk enough already	4.912	-	-	-	-	-	-	-	-	-	-	-	-
I get plenty of exercise by other means	-	-	-	-	-	-	-	-	-	0.403	-	-	-
Health													
I’m not in good health	-	-	20.730	13.474	-	-	0.287	-	-	-	-	1.818	-
I have a physical disability that affects my ability to walk	-	-	10.141	6.292	-	-	-	0.338	-	0.395	-	-	-
I have an injury that affects my ability to walk	-	-	-	-	-	2.312	-	0.356	-	-	-	-	-
Neighborhood Safety													
I don’t have a safe place to walk because of crime	-	-	11.261	4.181	-	-	0.119	-	0.339	-	-	-	1.191
Streets have poor lighting at night	-	2.710	-	-	2.226	-	-	-	-	-	-	-	-
I don’t feel safe walking alone at night	-	-	-	-	7.630	-	-	-	-	-	-	0.597	-
Time Availability													
I don’t have time	-	-	-	-	-	-	-	2.410	-	-	-	-	-
I don’t make time	-	-	-	-	-	-	-	1.902	-	-	-	1.708	-
Traffic Safety													
There is too much traffic in my neighborhood	-	2.413	-	-	-	-	-	-	-	-	-	1.673	-
Drivers often exceed the posted speed limits in my neighborhood	-	-	-	2.596	2.021	-	-	-	-	-	-	-	-
There are dangerous crossings in my neighborhood	-	3.577	-	-	-	-	-	-	-	-	-	-	-
Walking Preference													
I don’t like to walk	-	3.816	-	-	-	-	3.430	-	-	-	-	-	-
I don’t feel like walking	-	-	-	-	-	2.332	-	-	-	-	-	-	-
I have no one to walk with	-	5.491	-	2.495	-	-	-	-	-	-	-	-	-
Routes are boring	-	-	-	-	3.406	-	-	-	-	-	2.194	-	-

#### 3.2.2. Income Status

The second socio-demographic variable, household income, compares low and middle income with high income. Three potential barriers to walking are found to be related to higher agreement for poor and middle income households compared to high income households: *not in good health*, *have a physical disability*, and *no safe place to walk because of crime*. The first two barriers are related to quality of life. Households with lower income cannot access healthy foods and physical activity infrastructure as easily as medium and high incomes, thus increasing health problems and increasing the impact of disability [42]. Issues of crime can also be minimized by the affluent as they have more choice as to the neighborhood in which they live. Lower income households may have a much more limited neighborhood choice. Crime concerns have a much larger impact on the poor as perceptions of crime may be closer to reality than those perceived by members of middle income households.

#### 3.2.3. Female

The third socio-demographic variable examined is sex, comparing females to males. Seven potential barriers to walking are found to be related to higher agreement for females than males: *streets have poor lighting at night*, *I don’t feel safe walking alone at night*, *drivers often exceed the speed limit*, *I don’t like getting hot and sweaty*, *I don’t like being exposed to the sun*, *destinations are too far away*, and *routes are boring*. Concerns about walking at night due to poor lighting and other safety concerns are a common barrier for women [18,23,24,25], as women are the most likely population group to be victims of crime. Many of the other barriers are related to the aesthetics and their enjoyment of a trip, suggesting that when women perceive routes to be interesting they are more motivated to walk compared to men.

#### 3.2.4. Marital Status

The fourth socio-demographic variable examined is marital status, comparing single people to those who are married or cohabitate. Two potential barriers to walking are found to be related to higher agreement among those single compared to those who have a partner: *having an injury* and *not feeling like walking*. Having injuries that are not disabilities are generally temporary ailments that change the personal travel plans of those who agree. Not feeling like walking is found to affect those who are single more than married or cohabitating couples; this may be a result of such couples providing intrinsic motivation or encouragement to each other to be active, which is not readily available to those who are single.

#### 3.2.5. Student Status

The fourth socio-demographic variable examined is student status, comparing students to non-students. Two potential barriers to walking are found to be related to higher agreement for students than non-students: *I don’t like to get hot and sweaty* and *I don’t like to walk*. Students may identify more with not getting hot and sweaty because they are young and may be more concerned about their appearance than non-students. The preference of not liking to walk may be born out of a lack of car ownership of young people, which forces them to walk and take public transit.

Two potential barriers to walking are found to be related to higher agreement for non-students than students: *I’m not in good health* and *I don’t have a safe place to walk because of crime.* Students are often young and healthy, thus those who are not students may have health problems that do not impact students. Non-students also are concerned with crime more than students, which may be a result of students living in “student ghettos” located close to a university or college. These areas are places where there is a community and most of the residents are affiliated with the university or college.

#### 3.2.6. Employment Status

The fifth socio-demographic variable examined is employment status, comparing workers to non-workers. Two potential barriers to walking are found to be related to higher agreement for workers than non-workers: *I don’t have time* and *I don’t make time*. These barriers are related to the time constraints faced by workers. Two potential barriers to walking are found to be related to higher agreement for non-workers than workers: *having a physical disability* and *having an injury*. Both of these barriers would make working quite difficult, so the significance they have for non-workers is expected.

#### 3.2.7. Parenthood

The sixth socio-demographic variable examined is parenthood, comparing parents to non-parents. One potential barrier to walking is found to be related to higher agreement for non-parents than parents: *I don’t have a safe place to walk because of crime*. While parents may self-select a neighborhood where they live based on being safe and crime-free, child-free households may not have the same priorities.

#### 3.2.8. Driver’s License Ownership

The seventh socio-demographic variable examined is driver’s license ownership, comparing those owning a license to those not owning a license. Walking is required for those who do not have a license. Still, there are potential barriers that prevent those without a license from walking more, including *I get plenty of exercise by other means* and *I have a physical disability*. The first barrier suggests that those without a driver’s license are generally active people who complement their utilitarian walking with other types of physical activities. These people may believe that given their current levels of physical activity, it is unnecessary to walk more often. The second barrier suggests that those without a driver’s license are more likely to have a physical disability than those with a driver’s license. The disability may in fact prevent them from owning a driver’s license. The physical disability itself impacts the amount of walking that can be undertaken by those without a driver’s license. Such people are particularly vulnerable to social exclusion given their mobility constraints.

#### 3.2.9. Transit Pass Ownership

The eighth socio-demographic variable examined is transit pass ownership, comparing those owning a transit pass to those not owning a transit pass. One potential barrier to walking is found to be related to higher agreement for those with a pass than without a pass: *routes are boring*. This suggests that the routes taken for those who use transit regularly may prevent them from walking more often, although those who use public transit are forced to walk to and from stops which provides some opportunity to be active.

#### 3.2.10. Body Mass Index

The ninth socio-demographic variable examined is BMI. Five potential barriers to walking are found to be related to higher agreement when BMI increases: *I don’t like to get hot and sweaty*, *I’m not in good health*, *I don’t like being exposed to the sun*, *I don’t make time*, and *there is too much traffic in my neighborhood*. As high BMI is correlated with low levels of physical activity, there are many barriers expected to be agreed upon by this sub-group of the population. In particular, not being in good health is a common problem among those who are overweight or obese. Similarly, those who have high BMI may also not like to be active, which results in higher agreement in preference barriers, such as not liking to be exposed to the sun and not making time for walking. One potential barrier to walking is found to be related to higher agreement when BMI decreases: *I don’t feel safe walking alone at night*. Having a higher BMI can increase a person’s feeling of safety, as larger body types are more able to protect themselves when dangerous situations arise.

#### 3.2.11. Walkability Index

The final variable examined is the walkability index, comparing how potential barrier agreement relates to increasing walkability. Two potential barriers to walking are found to be related to higher agreement: *I don’t like to get hot and sweaty* and *I don’t have a safe place to walk because of crime*. The first barrier suggests that even in highly walkable neighborhoods, people’s comfort can influence the amount of walking they do. The second barrier suggests that walkable neighborhoods in Hamilton may be located in areas of the city where crime is more of a concern. As the walkability decreases, so does the concern of crime.

## 4. Conclusions

This study has investigated potential barriers to walking in Hamilton, Ontario, Canada using a descriptive analysis of the barriers followed by a series of binary logit models. Examining how sub-groups of the population associate with barriers to walking can provide policy makers with profiles of people who are potentially affected by each barrier. This also allows policy decisions to be made targeting specific population groups.

The results find that some barriers to walking have a larger impact on sub-groups of the population. While the Canadian public health system provides free health care to all Canadians, concerns with health barriers to walking are still more prevalent among the poor, middle class, and unemployed. This may be a result of the higher income and employed being able to afford the additional services provided by health benefit packages, such as physiotherapy and drug plans. The higher income are also able to afford health promotion activities, such as a gym membership and participation in sports, which all cost money. By working with the less fortunate, policy makers and health care providers can identify activities that can improve the perceived health of residents to minimize the impact health has on the ability to walk.

Issues of safety are a concern to the vulnerable sub-groups of women and senior citizens. Those who are vulnerable will have more fear of their neighborhood due to crime and pedestrian safety than the average citizen. While this is not a new finding [18,23,24,25], it is important to identify as policy makers need to do a better job of increasing safety in dangerous areas in the city.

Finally, those who are employed are far more likely to be influenced by barriers due to time availability. The amount of time reserved for working makes it difficult for workers to walk. Time barriers are reinforced by the ever increasing distance between home and work caused by urban sprawl, which limits the ability to walk to work even when it is desired. In order to minimize the influence work has on walking it is important for health promoters to highlight walking for other purposes, such as shopping or visiting friends, or walking during the work day, such as during lunch. Even though work may not be accessible, there are many other destinations that are far easier to reach on foot.

There are a few limitations to this study that should be considered when interpreting the results. The sample size of our study is small, thus limiting the number of barriers that could be examined using our methodology. Despite the small sample size, this random sample collected as part of the HALStudy is representative of Hamilton’s neighborhoods. This study also did not address directly how the barriers impact the actual walking behavior of the participants. Instead, it examined how sub-groups of the population, defined by socio-demographic variables, are associated with barriers in a multivariate framework so that health promotion experts and policy makers can find ways to minimize barriers for each group in order to increase walking of the entire population.

Future work examining walking should focus on how walking fits into the greater physical activity framework in Hamilton, Ontario, Canada. By understanding overall physical activity participation, it is possible to determine if those who do not participate in walking participate in any other types of physical activity. Future work should also consider how these barriers affect actual walking behavior measured in terms of distance, time, or number of such episodes.

## References

[1] U.S. Department of Health and Human Services (1996). Physical Activity and Health: A Report of the Surgeon General.

[2] Katzmarzyk P.T. (2011). The economic costs associated with physical inactivity and obesity in Ontario. Health Fit. J..

[3] Bassett D.R., Pucher J., Buehler R., Thompson D.L., Crouter S.E. (2008). Walking, cycling, and obesity rates in Europe, North America, and Australia. J. Phys. Act. Health.

[4] Gilmour H. (2007). Physically active Canadians. Health Rep..

[5] Rafferty A.P., Reeves M.J., McGee H.B., Pivarnik J.M. (2002). Physical activity patterns among walkers and compliance with public health recommendations. Med. Sci. Sports Exerc..

[6] Tremblay M.S., Warburton D.E.R., Janssen I., Paterson D.H., Latimer A.E., Rhodes R.E., Kho M.E., Hicks A., Leblanc A.G., Zehr L. (2011). New Canadian physical activity guidelines. Appl. Physiol. Nutr. Metab..

[7] Cragg S., Camerson C., Craig C.L. (2006). 2004 National Transportation Survey.

[8] Saelens B.E., Sallis J.F., Black J.B., Chen D. (2003). Neighborhood-based differences in physical activity: An environmental scale evaluation. Res. Pract..

[9] Pucher J., Dijkstra L. (2003). Promoting safe walking and cycling to improve public health: Lessons from The Netherlands and Germany. Am. J. Public Health.

[10] Brownson R.C., Hoehner C.M., Day K., Forsyth A., Sallis J.F. (2009). Measuring the built environment for physical activity: state of the science. Am. J. Prev. Med..

[11] Frank L.D., Sallis J.F., Saelens B.E., Leary L., Cain K., Conway T.L., Hess P.M. (2010). The development of a walkability index: Application to the neighborhood quality of life study. Br. J. Sports Med..

[12] Frank L.D. (2005). Multiple impacts of the built environment on public health: Walkable places and the exposure to air pollution. Int. Reg. Sci. Rev..

[13] Frank L.D., Sallis J.F., Conway T.L., Chapman J.E., Saelens B.E., Bachman W. (2006). Many pathways from land use to health: Associations between neighborhood walkability and active transportation, body mass index, and air quality. J. Am. Plan. Assoc..

[14] Herbert L., Owen V., Pascarella L., Streisand R. (2013). Text message interventions for children and adolescents with type 1 diabetes: a systematic review. Diabetes Technol. Ther..

[15] Ball K., Bauman A., Leslie E., Owen N. (2001). Perceived environmental aesthetics and convenience and company are associated with walking for exercise among Australian adults. Prev. Med..

[16] Hohepa M., Scragg R., Schofield G., Kolt G.S., Schaaf D. (2007). Social support for youth physical activity: Importance of siblings, parents, friends and school support across a segmented school day. Int. J. Behav. Nutr. Phys. Act..

[17] Mota J., Gomes H., Almeida M., Ribeiro J.C., Santos M.P. (2007). Leisure time physical activity, screen time, social background, and environmental variables in adolescents. Pediatr. Exerc. Sci..

[18] Timperio A., Ball K., Salmon J., Roberts R., Giles-Corti B., Simmons D., Baur L.A., Crawford D. (2006). Personal, family, social, and environmental correlates of active commuting to school. Am. J. Prev. Med..

[19] Brodersen N.H., Steptoe A., Williamson S., Wardle J. (2005). Sociodemographic, developmental, environmental, and psychological correlates of physical activity and sedentary behavior at age 11 to 12. Ann. Behav. Med..

[20] Cervero R., Duncan M. (2003). Walking, bicycling, and urban landscapes: Evidence from the San Francisco Bay Area. Am. J. Public Health.

[21] Copperman R.B., Bhat C.R. (2007). An analysis of the determinants of children’s weekend physical activity participation. Transportation.

[22] Clark A.F., Scott D.M., Yiannakoulias N. (2013). Examining the relationship between active travel, weather, and the built environment: A multilevel approach using a GPS-enhanced dataset. Transportation.

[23] Brownson R.C., Baker E.A., Housemann R.A., Brennan L.K., Bacak S.J. (2001). Environmental and policy determinants of physical activity in the United States. Am. J. Public Health.

[24] Gómez J.E., Johnson B.A., Selva M., Sallis J.F. (2004). Violent crime and outdoor physical activity among inner-city youth. Prev. Med..

[25] McMillan T.E. (2007). The relative influence of urban form on a child’s travel mode to school. Transp. Res. Part. A Policy Pract..

[26] Troped P.J., Saunders R.P., Pate R.R., Reininger B., Ureda J.R., Thompson S.J. (2001). Associations between self-reported and objective physical environmental factors and use of a community rail-trail. Prev. Med..

[27] King A.C., Castro C., Wilcox S., Eyler A.A., Sallis J.F., Brownson R.C. (2000). Personal and environmental factors associated with physical inactivity among different racial-ethnic groups of U.S. middle-aged and older-aged women. Health Psychol..

[28] Adachi-Mejia A.M., Drake K.M., MacKenzie T.A., Titus-Ernstoff L., Longacre M.R., Hendricks K.M., Beach M.L., Dalton M.A. (2010). Perceived intrinsic barriers to physical activity among rural mothers. J. Womens Health.

[29] Dawson J., Hillsdon M., Boller I., Foster C. (2007). Perceived barriers to walking in the neighborhood environment: A survey of middle-aged and older adults. J. Aging Phys. Act..

[30] Spinney J., Millward H. (2010). Time and money: A new look at poverty and the barriers to physical activity in Canada. Soc. Indic. Res..

[31] Clark A.F., Scott D.M. (2013). Does the social environment influence active travel? An investigation of walking in Hamilton, Canada. J. Transp. Geogr..

[32] Booth M.L., Owen N., Bauman A., Clavisi O., Leslie E. (2000). Social-cognitive and perceived environment influences associated with physical activity in older Australians. Prev. Med..

[33] Harrison R.A., Gemmell I., Heller R.F. (2007). The population effect of crime and neighbourhood on physical activity: an analysis of 15,461 adults. J. Epidemiol. Community Health.

[34] Owen N., Cerin E., Leslie E., duToit L., Coffee N., Frank L.D., Bauman A.E., Hugo G., Saelens B.E., Sallis J.F. (2007). Neighborhood walkability and the walking behavior of Australian adults. Am. J. Prev. Med..

[35] Statistics Canada (2012). Low Income Lines, 2010 to 2011.

[36] Cleland V., Ball K., Hume C., Timperio A., King A.C., Crawford D. (2010). Individual, social and environmental correlates of physical activity among women living in socioeconomically disadvantaged neighbourhoods. Soc. Sci. Med..

[37] Kuczmarski R.J., Ogden C.L., Guo S.S., Grummer-Strawn L.M., Flegal K.M., Mei Z., Wei R., Curtin L.R., Roche A.F., Johnson C.L. (2002). 2000 CDC growth charts for the United States: Methods and development. Vital Health Stat..

[B38-ijerph-13-00179] 38.StataCorp STATA Statistics/Data Analysis 2011.

[39] Harrell F. (2001). Regression Modeling Strategies.

[40] Stock J.H., Watson M.W. (2008). Heteroskedasticity-robust standard errors for fixed effects panel data regression. Econometrica.

[41] Davies H.T., Crombie I.K., Tavakoli M. (1998). When can odds ratios mislead?. BMJ.

[42] Larsen K., Gilliland J., Hess P.M. (2012). Route-based analysis to capture the environmental influences on a child’s mode of travel between home and school. Ann. Assoc. Am. Geogr..

